# Coldharbour Farm—The First Five Years
*A paper read to the Seventh International Congress of the World Federation for Mental Health, London, August, 1968.


**Published:** 1969-04

**Authors:** W. Alan Heaton-Ward

**Affiliations:** Consultant Psychiatrist-in-Charge, Stoke Park Hospital, Bristol


### COLDHARBOUR FARM — THE FIRST FIVE YEARS *

BY

W. ALAN HEATON-WARD, M.B., CH.B., D.P.M.

*Consultant Psychiatrist-in-Charge, Stoke Park Hospital, Bristol.*

### INTRODUCTION

Coldharbour was originally a farm house, and is situated about half a mile from Stoke Park Hospital for the mentally subnormal in Bristol, England. For many years it accommodated twenty-two of the older, female patients. In 1961, these patients were moved into Bristol and Coldharbour appeared to be ideal for a unit for fifteen disturbed adolescents. It was sufficiently near to the main hospital to make use of its services when necessary and yet sufficiently remote to maintain the distinction between the two different types of patients.

### AIM

The aim of the unit was to rehabilitate girls between about 15 and 25 years old, with I.Q.'s of about 70 and over, whose extremely disturbed behaviour had failed to respond to various authoritarian regimes, which included approved schools and prisons. The method was to be, as far as possible, permissive and psycho-therapeutically orientated. It was hoped that, ultimately, it would function completely independently of the main hospital as regards catering and that, as part of their training, patients would be taught to manage the housekeeping themselves—preparing shopping lists, purchasing food, and cooking it themselves. For various administrative reasons, this has not yet proved possible, but patients take it in turns to prepare some of the meals and to do the general housework as they would in their own homes.

### NURSING STAFFING

Initially, the unit was staffed by a ward-sister and a state-enrolled nurse during the day, and a staff nurse at night. The sister was married to a schoolmaster and had two teenage sons. Her husband voluntarily undertook teaching the girls in his spare time, and her sons took part in the recreational activities in the unit. This excellent arrangement terminated in 1963, when, unfortunately, the sister obtained a post elsewhere. At this time a state-enrolled nurse took over night duty.

The nursing establishment remained the same until 1964, when an additional sister was appointed to the unit for day duty. Student nurses spend time in the unit as part of their training. Staff in the unit are changed as rarely as possible, but unavoidably, at times of holidays or sickness, temporary replacements have to be found. It has been noticeable that difficulties arise in the unit at these times, when patients test out any new staff on arrival.

* A paper read to the Seventh International Congress of the World Federation for Mental Health, London, August, 1968.

### THE PATIENTS

During the five years between 16th March, 1961 and 15th March, 1966 there were thirty-one direct admissions to Coldharbour and nine transfers of patients who had already spent considerable periods in other units in the Stoke Park Hospital Group. This paper is concerned only with the direct admissions. (For this purpose, three patients who had spent up to six weeks elsewhere in the group are considered as direct admissions).

In spite of the apparent national shortage of beds for disturbed adolescents, the bed occupancy of Coldharbour has remained low until recently and there have never been more than thirteen patients resident at one time. During the first year, seven patients were admitted and during the remaining four years, ten, six and two patients respectively. Whenever possible admissions have been spaced so as to allow new admissions to be absorbed into the community one at a time. The low figure for 1966 was due to restriction of admissions during a particularly difficult period of patient instability, at a time of shortage of medical staff even more acute than usual.

The patients' ages on admission ranged from 13 years 11 months to 25 years (average 18 years 6 months) and their I.Q.'s from 57 to 107 (average 74.7). Twenty-four were admitted from the South Western Regional Hospital Board's catchment area and seven from outside this area (mainly from the London area—one hundred and twenty miles away). Twenty-four were referred by consultant psychiatrists, six by local authority mental health departments and one by a children's department.

Examination of the patients' backgrounds revealed the familiar unsatisfactory pattern in most cases. Nine of the patients were illegitimate. Of the others, the father was known to be alive in fifteen cases and the mother in seventeen cases. In only ten cases were both natural parents living together. In three of the other cases the parents were divorced, and in two of these cases, one or other of the natural parents had remarried and was living with the stepfather or stepmother. Six of the patients had been adopted, but both adoptive parents were known to be alive in two cases only. In twenty-six of the cases the patient had an unsatisfactory relationship with the parents or adoptive parents.

History of other possibly significant ætiological factors was available in only seven cases. In two, there was a family history of mental subnormality, in one, a history of head injury at birth, in another a history of head injury in childhood, in two, a history of cerebral infection and in one, a history of both head injury and cerebral infection.

Twenty-two of the patients had attended secondary modern schools, two had attended primary schools only, and the remaining seven, schools for the educationally subnormal. Before admission to Coldharbour only four had a satisfactory employment record. Of the remainder, seven had a fairly satisfactory work record, eleven an unsatisfactory record and nine had never been employed at all.

Sixteen patients were said to have behaved in a sexually promiscuous manner before admission to Coldharbour. Three had had illegitimate children before admission and two were pregnant at the time of admission. Two had been involved in incestuous relationships with father or a brother. One was married but separated from her husband.

Relationships with members of the patients' own sex outside the family were less frequently commented on in case histories before admission but in only five cases did they seem to be satisfactory.

Fourteen of the patients had a history of violence. Twelve had appeared before the Juvenile Courts, one before a higher Court and three before both. As a result, six had been to approved schools, eight had been in prison or a remand home and one in a special security hospital

Although, as mentioned, the unit itself was open and a permissive regime adopted as far as possible, we found it more satisfactory in most cases to request that patients should come into Coldharbour initially on an order under the Mental Health Act rather than informally or as a condition of probation, or as a condition of residence while a patient was in the care of a local authority Children's Department. Thirteen were admitted on hospital treatment orders, seven on Court orders, one was transferred on order of the Home Secretary and ten were admitted as informal patients not liable to compulsory detention.

### THE PROGRAMME

During the settling-in period patients are occupied in housework and in preparing meals. In this they are given instruction by nursing staff. The facilities of the main hospital are available to them and members of the occupational therapy and teaching staff visit Coldharbour as required. We have established a very good relationship with a local technical college where girls from Coldharbour can attend on a daily basis. Unfortunately, on each occasion we have arranged this, patients have discontinued attendance of their own accord because they are self-conscious of the difference in background between themselves and the other students attending the college, in spite of the most sympathetic attitude of staff and students alike.

Once a patient has achieved reasonable stability and satisfactory work habits within the unit, one of our social workers tries to obtain employment for her to which she can travel each day from Coldharbour. The range of employment includes domestic and laundry work in nearby general hospitals, domestic and laundry work in hotels, work in restaurants and canteens and various jobs in offices and factories.

Patients are encouraged to take their recreation away from the main hospital, either in Coldharbour itself or in town. Within the unit they follow the usual teenage pursuits—pop music, dancing, television. They are encouraged to have boy friends but are asked to invite them along to tea in the unit at the weekend, before going out with them. Patients can, if they wish, attend a local youth club and groups go to dances in Bristol from time to time.

With over 1700 other in-patients to care for, as well as numerous other responsibilities in hospital and the community, the three senior psychiatrists in the Stoke Park Hospital Group are unable to devote all the time they would wish to the in-patients at Coldharbour. Their role is, under these circumstances, largely to provide support for the nursing staff in their very difficult task of rehabilitating these patients. The medical and nursing staff give individual psychotherapy to patients but any success we may achieve at Coldharbour is really dependent on the nurses' ability to make a satisfactory relationship with these girls, perhaps for the first time in their lives. There is no formal group psychotherapy, but informal discussion groups in the evening between patients and staff serve a similar purpose.

After a period of stability and satisfactory daily employment, attempts are made to find residential employment and lodgings for patients. It is often not easy to find suitable lodgings with landladies who understand and are tolerant of these girls.

### THE RESULTS

During the period under review, the duration of patients' stay varied from two to sixty-one months, the average duration of stay being 20.4 months. As could only be expected, patients continued their pre-admission pattern of behaviour for varying periods after admission. Thus, fifteen patients were violent to other patients and eight patients were violent to staff on one or more occasion. Five were destructive. Six were known to be sexually promiscuous and five were suspected of this. However, as far as we were aware, none became pregnant during their stay at Coldharbour in the period under review. Twenty of the patients absconded on at least one occasion.

Even in a permissive unit, there are limits to the behaviour which can be tolerated from consideration of the welfare of the patient herself, of the other patients and, not least, of the staff, and on occasions it became necessary to transfer fifteen patients to the main hospital under closer supervision for varying periods of time. In the case of two patients their behaviour became so unmanageable that they had to be transferred to a special security hospital.

Two patients were still in the unit at the end of the period under review, but I have attempted an assessment of the success or failure of our efforts in the case of others, based on the progress reports of the nursing staff. On this basis I estimate that the rehabilitation was satisfactory in nine cases, fairly satisfactory in two cases, unsatisfactory in thirteen cases, and that the other five patients were in the unit for too short a period for an assessmen to be of any value.

To test the validity of my assessment, in November 1967 I asked the local authority of the area to which each patient was discharged to make their own assessment and to place the patient in one of the three categories—'satisfactory', 'unsatisfactory', 'precarious'.

Of the nine cases classified by me as 'satisfactory' on leaving Coldharbour, local authorities placed two in the 'satisfactory' category, classified one as 'doubtful satisfactory', one as 'unsatisfactory' and one as 'precarious'. They classified the 'fairly satisfactory' patient as 'satisfactory'. They were unable to trace the whereabouts of two of the patients and, unfortunately, did not place the remaining two patients in any category. Adopting the same criteria I used in making my original assessment, I placed the two latter patients in the 'satisfactory' category on the information in the local authority reports.

Of the thirteen cases classified by me as 'unsatisfactory' on leaving Coldharbour, the local authorities placed only two in the 'unsatisfactory' category, five in the 'precarious' category, one in the 'satisfactory' category and did not classify three at all. They were unable to trace the whereabouts of two other patients. Using the criteria referred to above, I placed one of the other patients unclassified by the local authority in the 'satisfactory' category and two in the 'unsatisfactory' category.

In summary, it can be said that of the patients whose whereabouts were known, eight appeared to have made satisfactory progress since leaving Coldharbour for periods ranging from one year six months to four years eight months (mean two years nine months). How far this improvement may be due to our efforts at Coldharbour is, of course, a matter of speculation and twenty-five per cent may, in any case, appear a low success rate. However, it was achieved in patients who had previously defeated all other efforts to help them and we know it could be so much higher if the establishment was adequate to enable psychiatric and other staff to devote more time to the patients in Coldharbour.

*Acknowledgements*

I wish to thank Drs. J. Jancar and W. H. K. Carpenter for permission to include in this paper reference to patients under their care, hospital medical staff and local authority medical and child-care staff for providing follow-up reports and Mrs. V. Curtis for typing the manuscript.

